# Predictive factors for dental inflammation with exacerbation during cancer therapy with FDG-PET/CT imaging

**DOI:** 10.1007/s00520-020-05909-9

**Published:** 2021-01-07

**Authors:** Mai Kim, Trang Thuy Dam, Masaru Ogawa, Takahiro Shimizu, Takahiro Yamaguchi, Keisuke Suzuki, Takuya Asami, Jun Kurihara, Satoshi Yokoo

**Affiliations:** 1 Department of Oral and Maxillofacial Surgery, and Plastic Surgery, Gunma University Graduate School of Medicine, 3-39-22, Showamachi, Maebashi, Gunma 371-8511 Japan; 2 Department of Diagnostic Radiology and Nuclear Medicine, Gunma University Graduate School of Medicine, 3-39-22, Showamachi, Maebashi, Gunma 371-8511 Japan

**Keywords:** Oral adverse event, Fluorodeoxyglucose-positron emission tomography/computed tomography, PET accumulation of dental lesion grading, Dental inflammation exacerbation during cancer therapy, Cancer therapy

## Abstract

**Purpose:**

Oral adverse events, such as dental inflammation with exacerbation, are stressful and lead to poor nutrition in patients undergoing cancer therapy. Thus, the prediction of risk factors for dental inflammation with exacerbation is important before cancer therapy is initiated. We hypothesized that, during cancer therapy (DIECT), fluorodeoxyglucose-positron emission tomography/computed tomography (FDG-PET/CT) imaging could be useful to predict dental inflammation with exacerbation.

**Methods:**

We enrolled 124 patients who underwent FDG-PET/CT for diagnostic staging before cancer treatment. We then assessed DIECT outcomes after basic perioperative oral treatment. Moreover, we evaluated clinical parameters, therapeutic strategies, periodontal examination (probing depth (PD) and bleeding on probing (BOP)), dental imaging, and FDG-PET/CT imaging results of patients with and without DIECT. Furthermore, PET/CT images were assessed as per the FDG accumulation of the dental lesion (PAD) grading system.

**Results:**

Univariate analysis demonstrated significant differences in age, periodontal examination (PD and BOP), and PAD grade between patients with and without DIECT. Furthermore, multivariate logistic regression analysis identified independent predictive factors for a positive periodontal examination (PD) (odds ratio (OR) 5.9, 95% confidence interval (CI) 1.8–19.7; *P* = 0.004) and PAD grade (OR 11.6, 95% CI 3.2–41.2; *P* = 0.0002). In patients with cancer, PAD grade using FDG-PET/CT imaging was an independent and informative risk factor for DIECT.

**Conclusion:**

Our results suggested that, for patients with DIECT, periodontal examination and PAD grade were independent predictive factors. Hence, regardless of the presence or absence of any lesion on dental imaging, PAD grade might be an additional tool, in addition to periodontal examination that potentially improves oral care management.

## Introduction

Recently, because of the number of survivors and years of survival after treatment, advances in cancer therapeutic techniques have increased [1]. Although cancer treatments, such as surgery, radiotherapy, and systemic therapy, can control cancer, adverse events that occur during treatment remain challenging. Systemic adverse events such as pneumonia and sepsis are acute and life-threatening [2–5]. Moreover, local adverse events should be carefully considered because they may interfere with anti-cancer treatment completion. Therefore, supportive care is extremely important for the success of cancer treatment; furthermore, appropriate supportive treatment for adverse events must be performed in a timely manner.

Among local adverse events, multiple oral complications always cause a lack of motivation for disease control [6]. Oral complications related to cancer treatment depend on the patient’s primary tumor and the type of treatment. These oral complications include functional limitations and neurological problems associated with surgical treatment [7]. Common oral complications associated with chemotherapy and radiation include oral mucositis; infections with bacteria, fungi, or viruses; salivary gland dysfunction or xerostomia; taste and functional disorder; and poor nutrition [8, 9]. These oral complications affect the quality of life of patients with cancer. Carvalho et al. [10] reported that health professionals involved in oral healthcare should assess the oral health and function of patients with cancer, thus enabling the prevention or treatment of oral complications caused by oncological treatment. A recent systematic review supported the evidence for the requirement to use multi-agent combination oral care protocols complemented by expert opinion to manage oral mucositis in cancer patients [11]. The recommendations of the Multinational Association of Supportive Care in Cancer support the importance of basic oral care in cancer. Thus, before initiating anti-cancer treatment, oral function should be sufficiently managed. In the past, evaluations after the onset of oral adverse events have been performed with the Oral Assessment Guide and the National Cancer Institute grading scale (CTCAE); however, evaluations for predicting oral adverse events have not yet been performed.

^18^F-fluorodeoxyglucose-positron emission tomography/computed tomography (FDG-PET/CT) is a recommended nuclear imaging modality for the diagnostic work-up, staging, treatment response evaluation, and prediction of outcome in several types of cancer [12]. However, the role of FDG-PET/CT in the assessment and prediction of oral adverse events remains unclear. Therefore, in this study, we examined whether FDG-PET/CT imaging before the initiation of cancer treatment could be a predictive tool of dental inflammation with exacerbation during cancer therapy (DIECT) in oral care management.

## Patients and methods

### Study population, participants, and period

This study was approved by the institutional research board (IRB number: HS2018-190). We retrospectively evaluated 124 patients who underwent FDG-PET/CT for diagnostic cancer staging before cancer treatment at Gunma University Hospital between April 2016 and October 2018. This study enrolled patients with cancer who were consulted for perioperative oral care management. Informed consent was obtained from patients for their involvement in this study.

### Extraction of variables

Observation variables included the patients’ physical factors, treatment factors, systemic adverse events (aspiration pneumonia), clinical periodontal examination (probing depth (PD) and bleeding on probing (BOP)), and imaging findings (dental imaging included orthopantomography or dental X-ray imaging and FDG-PET/CT).

Clinical periodontal examinations were based on the 2015 Japanese Society of Periodontology Clinical Practice Guideline for Periodontal Treatment (http://www.perio.jp/publication/upload_file/guideline_perio_plan2015_en.pdf). Periodontal examination was performed by probing depth measurement at determining one point for each tooth to assess the severity of inflammation in this study. The grading, based on clinical periodontal examination, was as follows: grade 1, mild periodontitis (probing depth, < 4 mm or no teeth); grade 2, moderate periodontitis (probing depth, ≥ 4–< 6 mm); and grade 3, severe periodontitis (probing depth, ≥ 6 mm). BOP refers to one probing per tooth; to express the bleeding index as a percentage, it is assessed by periodontal tissue inflammation that is divided by the total number of available teeth in the mouth and multiplied by 100.

Imaging results using orthopantomography or dental X-ray imaging were evaluated based on the degree of tissue destruction as follows: class I, mild periodontitis with bone resorption (bone level (BL)) or attachment loss (AL) of < 30% of root length and no furcation involvement; class II, moderate periodontitis with BL or AL of 30–50% and furcation involvement; and class III, severe periodontitis with BL or AL of ≥ 50% and involvement of class II or III furcation. The periapical lesions were diagnosed as any periapical radiolucency that was > 4 mm in size [11, 13].

**Fig. 1:**
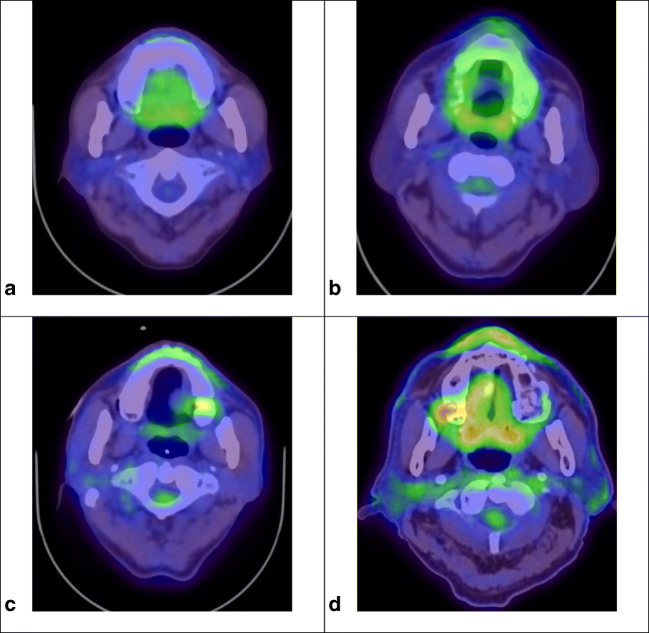
The PAD scoring system was defined as follows: grade 0, no accumulation; grade 1, non-specific accumulation; grade 2, specific or spot shape accumulation; and grade 3, abnormal PET characteristic uptake for dental lesions

FDG-PET/CT image analysis was performed by attenuation-corrected PET data using a *syngo*.via® (SIEMENS Healthcare©, Erlangen, Germany) device and an oncology software package (SIEMENS Healthcare©, Erlangen, Germany). An independent dental radiologist (10-year experience), who was blinded to the clinical status of the patient, analyzed all PET images. FDG-PET/CT imaging findings were then visually evaluated and classified into a PET accumulation of dental lesion (PAD) scoring system. The PAD scoring system is shown as follows: grade 0, no accumulation; grade 1, non-specific accumulation; grade 2, specific or spot shape accumulation; and grade 3, abnormal PET characteristic uptake for dental lesions (Fig. 1).

### The protocol of perioperative oral treatment at Gunma University

Patients scheduled for cancer treatment were referred to our department by the attending physician, and periodontal examination and orthopantomography or dental X-ray imaging was used for diagnosing oral lesions. First, the dentist recommended a treatment plan evaluating the progression of periodontal disease and oral diseases that may interfere with cancer treatment. Second, acute oral infections such as caries and periodontal and endodontic diseases were identified and treated before cancer treatment. After the complete treatment of infections and bacteria plaque, the patient received instructions on oral hygiene. Finally, prior to the initiation of cancer treatment, the dentist reassessed the patients’ oral cavity to ensure major issues that could adversely affect cancer therapy were resolved. During cancer treatment, patients who complained about pain in the oral cavity were considered as DIECT-positive. The patients with DIECT were treated with additional periodontal care; in this study, the endpoint was DIECT with cancer therapy.

### Statistical analysis

For univariate analysis, the objective variable was set as DIECT, and the explanatory variables included age, gender, smoking, diabetes, primary site, TNM staging, operation time, presence or absence of surgical therapy, radiation therapy, chemotherapy, unknown fever during treatment, periodontal examination (PD and BOP), orthopantomography or dental X-ray imaging, periapical lesions, and PAD score. For explanatory variables indicating continuous values, we used a Mann–Whitney *U* test; for binary variables, a cross-tabulation table was created, and a chi-square independent test was performed. A multivariate Cox proportional hazards model analysis was performed to determine the significant odds ratios (ORs) for DIECT. The variables for significant differences in univariate analysis were selected as explanatory variables; moreover, logistic regression analysis was performed using the variable increase method DIECT as the objective variable. Before the multivariate analysis, variables were examined for a correlation coefficient of |*r*| > 0.7. Statistical analyses were then performed using SPSS (IBM SPSS ver.25, Armonk, NY, USA). For all tests, *P* < 0.05 was considered to be significant.

## Results

This study evaluated 124 patients with cancer who underwent surgery, chemotherapy, or radiotherapy with FDG-PET/CT. Table 1 lists the clinical characteristics of 124 enrolled patients, the clinical potential variables related to DIECT development, and the results of univariate analysis. The patient age quartiles were 58–71 and 63–76 years (median, 66.6 and 69.0 years) in the DIECT (*n* = 18) or without DIECT (*n* = 106) groups. Moreover, 50% of primary cancers were located in the head and neck area, and the number of patients with stage I/II and III/IV as per the TNM staging system was approximately equal. The most frequently requested departments were otorhinolaryngology and hepatobiliary-pancreatic surgery, followed by gastrointestinal surgery. There was an average of 30 days from PET examination until cancer therapy was initiated. The oral care intervention period lasted an average of 28 days. Among cancer therapy types, 60% of patients had surgery, and ~ 50% of patients had chemoradiotherapy. Clinically, aspiration pneumonia was diagnosed and treated in 6% of cases. DIECT was observed in 15% of patients during cancer treatment; these dental inflammations were apical periodontitis and acute periodontal disease.

**Table 1:** Patient characteristics and results of the univariate analyses with or without dental inflammation exacerbation (*n* = 124)

		DIECT (+) (*n* = 18)	DIECT (−) (*n* = 106)	*P* value
Physical factors	Age (years), (median, quartile)	66.5 (58–71)	69.0 (63–76)	0.04
	Gender (M:F)	11 (61%):7 (39%)	72 (68%):34 (32%)	0.57
	Smoking (±), *n* (%)	9 (50%):9 (50%)	58 (55%):48 (45%)	0.45
	Diabetes mellitus (±), *n* (%)	5 (28%):13 (72%)	32 (30%):74 (70%)	0.88
	Primary tumor site (head and neck/others), *n* (%)	7 (39%):11 (61%)	48 (45%):58 (55%)	0.61
	TNM staging (I, II/III, IV), *n* (%)	7 (39%):11 (61%)	50 (47%):56 (53%)	0.24
Therapeutic factors	Operation time (min), (median, quartile)	443 (233–576)	442 (374–533)	0.72
	Surgical therapy (±), *n* (%)	12 (66%):6 (34%)	47 (44%):59 (56%)	0.94
	Chemo therapy (±), *n* (%)	7 (39%):11 (61%)	32 (30%):74 (70%)	0.25
	Radiotherapy (±), *n* (%)	7 (39%):11 (61%)	40 (34%):66 (66%)	0.54
Dental examination	Periodontal examination (probing depth) (grades 1, 2/grade 3), *n* (%) Bleeding on probing (%), (median, quartile)	8 (44%):10 (56%) 21.8 (8.0–71.7)	86 (81%):20 (19%) 0 (0–36.8)	0.001 0.013
Imaging factors	Orthopantomography (class I, II/class III), *n* (%) Periapical lesions (%), (negative, 4 mm>)	9 (50%):9 (50%) 15 (83%): 3 (17%)	65 (61%):41 (39%) 97 (92%):9 (8%)	0.367 0.299
	PAD score (grade 0, 1/grade 2, 3), *n* (%)	5 (28%):13 (72%)	80 (76%):26 (24%)	< 0.001
Systemic adverse event	Aspiration pneumonia (±), *n* (%)	2 (11%):16 (89%)	5 (5%):101 (97%)	0.98

*DIECT* dental inflammation exacerbation during cancer therapy

Continuous variable: Mann–Whitney *U* test

Binary variable: chi-square test of independence

Regarding clinical periodontal examination, PD grades 1–2 was observed in 8 (44%) patients with DIECT and in 86 (81%) patients without DIECT. Moreover, 21.8% of patients presented with BOP, while patients without DIECT did not show BOP. Regarding PET imaging, PAD score grades 0–1 was observed in 5 (28%) patients with DIECT and 80 (76%) patients without DIECT.

Univariate analysis suggested that the potential variables were related to age, periodontal examination (PD and BOP), and PAD score (*P* = 0.04, *P* = 0.001, *P* = 0.013, and *P* < 0.001; Table 1). These variables determine whether there was a strong correlation with an absolute value of a correlation coefficient exceeding 0.7 between each explanatory variable before multivariate analysis. The stepwise selection procedure identified two of the variables as significant using univariate analysis such as age, periodontal examination (PD and BOP), and PAD score. Significant factors were identified for developing periodontal examination PD (OR 5.9, 95% confidence interval (CI) 1.8–19.7; *P* = 0.004) and PAD grade (OR 11.6, 95% CI 3.2–41.2; *P* < 0.001; Table 2).

**Table 2:** Logistic regression analysis results for variables extracted by the increase method

Factor	Case (n = 124)	DIECT (±)	Regression coefficient	Standard error	Odds ratio	95% CI		*P* value
Periodontal examination Probing depth (grade 1, 2)	94	8/86	1.7	0.6	5.9	1.8–19.7	0.004	
Probing depth (grade 3)	30	10/20						
PAD score grades 0, 1	94	8/86	2.4	0.6	11.6	3.2–41.2	0.0002	
PAD score grades 2, 3	30	10/20						

*DIECT* dental inflammation exacerbation during cancer therapy

### Representative cases

**Fig. 2:**
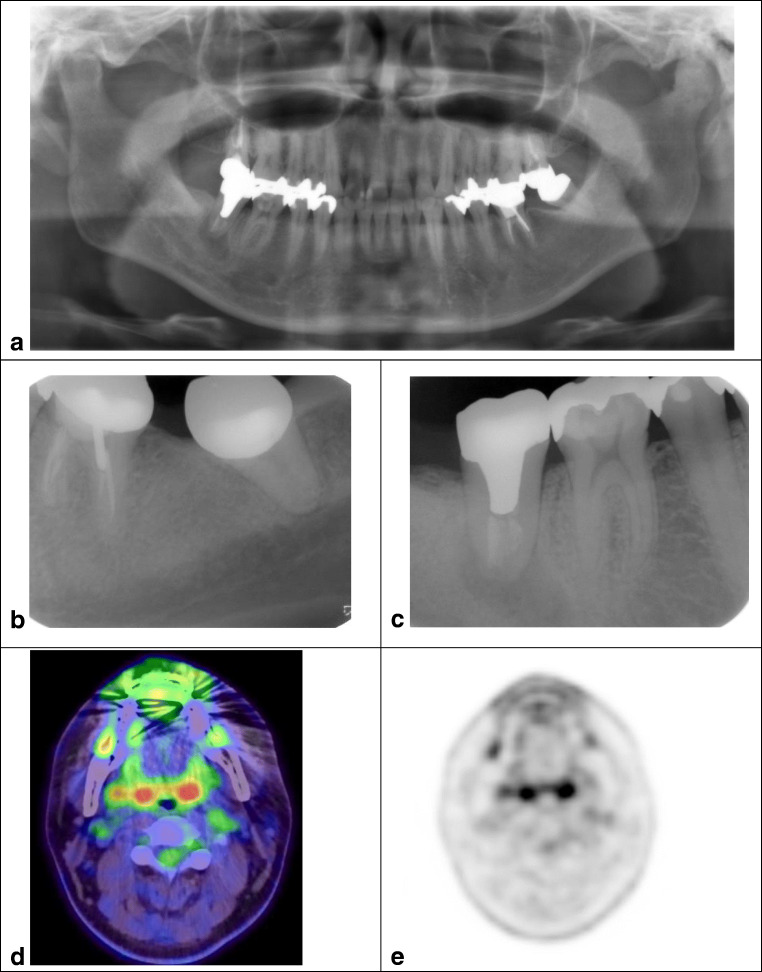
Representative cases. A 30-year-old male with external auditory canal cancer after receiving heavy ion therapy. He had negative clinical symptoms in the oral cavity when visiting our department. The lesion was found at the right mandibular second molar root apex. Panoramic radiography (**a**), dental radiography (**b**, **c**), and ^18^F-FDG-PET/CT with PAD score grade 3; right lesion (**d**, **e**)

Figure 2 shows certain representative cases in this study. Figure 2a shows a 30-year-old man with external auditory canal cancer after receiving heavy ion therapy. Although he had negative clinical symptoms in the oral cavity when visiting our department, FDG-PET/CT revealed a lesion at the right mandibular second molar root apex. Panoramic radiography (a), dental radiography (b, c), and ^18^F-FDG-PET/CT with a PAD score of grade 3 were performed; the right lesion is shown in d and e. DIECT was not observed before the start of cancer therapy. During heavy ion therapy, DIECT was observed; therefore, root canal treatment and periodontal treatment were required for these secondary retromolar teeth. Morphological radiographs, such as dental radiographic images, could not be evaluated as chronic or active inflammation lesions. Although FDG-PET images can be used to evaluate active inflammation, the specific FDG accumulation with oral and maxillofacial lesion suggested that active inflammation was confirmed in both lower secondary molars before the treatment was started. This served as a warning that active intervention in dental treatment was necessary before cancer therapy was started.

## Discussion

Because cancer treatment has made remarkable progress in recent years, the importance of supportive care for adverse events has been recognized [14–16]. To reduce adverse events and prevent infectious complications, supportive care during adverse events, such as prescription of antiemetic drugs and nutritional management, is important. Adverse events in the oral cavity are no exception, and oral mucositis and dental inflammation may make eating and talking difficult, thus consequently interrupting treatment and changing the outcome. Therefore, it is extremely important to evaluate dental inflammation before initiating cancer treatment.

Currently, periodontal diseases have primarily been evaluated via local examinations. Periodontal examination (PD and BOP) is known to be effective for tooth brushing index and patient self-care control [17]. However, PD and BOP, which rely on the evaluator’s probe pressure and the placement of the probe tip in the periodontal pocket, need to be considered for their reproducibility in repeated probing attachment level measurements [18]. However, their reproducibility might be limited because of differences in the evaluator’s method and the fact that the examination of each pocket and each tooth is tedious. Furthermore, although accurate, this examination is a complicated technique and can only be performed by professionals in dentistry, e.g., dentist or dental hygienist.

These results suggest that FDG accumulation in the oral region is useful as a predictive marker for dental inflammation. As characterized by FDG, when an inflammatory response is elicited in local tissues, leukocytes are activated and secrete cytokines. Locally collected inflammatory cells (granulocytes, lymphocytes, and macrophages) are activated by accumulation at a high density, and glucose consumption increases 50 times [19, 20]. Moreover, FDG does not accumulate in the central necrotic area; instead, it directly surrounds this section and accumulates in areas in which inflammatory cells, such as neutrophils, infiltrate. Furthermore, FDG infiltrates macrophages that spread outside the necrotic area. FDG is distributed in granulation tissue but does not accumulate in granulation tissue where fibroblasts are the center [20]. Because FDG is selectively accumulated by neutrophils and macrophages in inflamed tissues, it is possible to detect periodontal disease, and the pathogenesis of apical inflammatory lesions is reflected as the high accumulation of FDG. Thus, FDG accumulation could be an indicator of DIECT.

In clinical practice, PET imaging is performed to assess the staging of cancer before initiating the required treatment modality [21, 22]. Compared to periodontal examination, PET imaging outcomes are highly reproducible and not heavily influenced by the evaluator [23]. Furthermore, regardless of the presence or absence of inflammation in each tooth, periodontal examination can only evaluate the depth and activity of the periodontal pocket. However, PET can evaluate beyond the periodontal pocket and detect other tumors and metastases; moreover, the information on the degree of inflammation from PET images might be shared with other physicians.

Cancer-treated patients are at a high risk of a weakened immune system as an adverse event. A decrease in the number of neutrophils because of infection leads to symptoms such as fever; however, the causative pathogenic bacterium is unknown and difficult to clinically identify [24]. Multiple studies reported that, after allogeneic hematopoietic stem cell transplantation, periodontal infections may contribute to the increased risk of bacteremia during neutropenia [25, 26]. There are no standard criteria to treat asymptomatic chronic dental diseases before cancer therapy. Moreover, a delay in initiating cancer treatment for dental treatment poses a disadvantage for patients. Supplementing the dental evaluation with the PAD scoring system can potentially provide dental treatment priority, urgency, and triage of patients with an imminent treatment schedule. A clinical periodontal examination reflects the patient’s current oral condition but cannot predict the deterioration of oral environment in patients who are expected to have side effects associated with cancer treatment. Furthermore, orthopantomography or dental X-ray imaging provides morphological images that reflect the results of periodontal treatment up to the present time; however, the information is based on an earlier time point and does not aid in predicting future periodontal conditions.

To summarize, our study suggested that it is possible to evaluate the activity of inflammation by screening FDG accumulation in the oral region. Depending on the presence or absence of active inflammation, it is possible to provide a timing of dental treatment intervention and oral care considering the requirement for dental treatment. The results suggest the requirement for aggressive oral care in cases with the specific accumulation of FDG without clinical symptoms prior to treatment. Because the intraoral environment can be determined only by referring to image examinations taken for cancer therapy, FDG-PET can be expected as a new screening and assessment tool that can objectively observe intraoral results in multidisciplinary collaboration.

Evaluating FDG accumulation for oral and maxillofacial lesions is a helpful semi-quantitative and non-invasive method to assess and determine the treatment for chronic dental disease before initiating cancer treatment. PAD grading might be useful to screen for activity dental inflammation. Moreover, the verification for FDG uptake in oral and maxillofacial lesions using PET imaging is considered a highly versatile image evaluation that can effectively use limited medical and human resources.

Our study has several limitations. First, FDG accumulation for oral and maxillofacial lesions might reflect both dental inflammation and the possibility of neoplastic lesions, malignant tumors in the oral region, or bone metastases to the bone marrow of oral and maxilla facial lesion. Second, because of the retrospective method, the clinical symptoms of patients were evaluated based on their medical records, which did not include the visual analog score (VAS). Hence, in future studies, we plan to add VAS, which can be an index of objective evaluation and image inspection for evaluating periodontal pain or oral adverse events. Third, the PAD grade adds local periodontal inflammation, but does not address multiple other causes of inflammatory reaction that may occur during cancer therapy including mucositis, mucosal infection, and other oral complications of cancer therapy. Finally, the clinical periodontal examinations criteria in the JSP guideline (http://www.perio.jp/publication/upload_file/guideline_perio_plan2015_en.pdf) are not intended to be used for patients who are planned to receive cancer treatment. Therefore, an additional suitable clinical examination regarding periodontal disease for patients with cancer treatment should be investigated.

To summarize, the periodontal examination of PD and PAD grade were independent predictive factors for patients with DIECT. Hence, regardless of the presence or absence of any lesion detected on dental imaging, PAD grade on FDG-PET imaging might be an additional assessment tool, in addition to periodontal examination that potentially improves oral care management in patients with cancer.
